# Progress in Identifying Infants with Hearing Loss — United States, 2006–2012

**Published:** 2015-04-10

**Authors:** Tonya R. Williams, Suhana Alam, Marcus Gaffney

**Affiliations:** 1 Division of Human Development and Disability, National Center on Birth Defects and Developmental Disabilities, CDC

Congenital hearing loss affects one to three of every 1,000 live born infants (1) and negatively impacts children through delayed speech, language, social, and emotional development when undetected (2,3). To address this public health issue, jurisdiction-based Early Hearing Detection and Intervention (EHDI) programs are working to ensure all newborns are screened for hearing loss, receive follow-up diagnostic testing (DX) if they do not pass the screening, and are enrolled in early intervention (EI) services if diagnosed with a permanent hearing loss. Although substantial progress has been made in the provision and documentation of services, challenges remain because, unlike screening results, diagnostic test results and enrollment in EI are not consistently reported to the EHDI programs. Therefore, it is difficult for states and territories to know if infants received recommended follow-up services (diagnostic testing and/or EI services), often resulting in infants being classified at either stage as lost to follow-up (LFU)/lost to documentation (LTD). To assess progress toward identifying children with hearing loss and reducing LFU/LTD for DX (LFU/LTD-DX) and EI enrollment (LFU/LTD-EI*), CDC analyzed EHDI surveillance data for 2006–2012. Results indicated that the number of jurisdictions reporting data increased from 49 to 57, rates of screening increased from 95.2% to 96.6%, rates of referral from screening decreased from 2.3% to 1.6%, rates of diagnosis among infants not passing their final screening increased from 4.8% to 10.3%, and enrollment in EI among children diagnosed with hearing loss increased from 55.4% to 61.7%, whereas rates for both LFU/LTD-DX and LFU/LTD-EI declined. These findings show sustained progress toward screening, identification, and enrollment in EI as well as highlighting the need for continued improvements in the provision and documentation of EHDI services.

Data were gathered by using the EHDI Hearing Screening and Follow-up Survey (HSFS), which was fully implemented starting in 2006. This survey is sent annually to the EHDI program coordinator in each U.S. state, the District of Columbia, and each participating territory and freely associated state. The HSFS requests nonestimated, aggregate information about the receipt of hearing screening, diagnostic testing, and EI for every occurrent birth within the jurisdiction. The numbers of occurrent births are compared for accuracy with data from the jurisdiction’s Vital Records program and the National Vital Statistics System. Infants were classified as LFU/LTD-DX or LFU/LTD-EI if they did not receive recommended follow-up services or if they received services without the results being reported to the jurisdictional EHDI program. LTD can occur because the results of diagnostic testing and enrollment in EI are not universally required to be reported. Although strategies used to target LFU and LTD differ, these two categories are grouped together in the HSFS because it is problematic for most programs to differentiate between these different types of cases. The denominators for LFU/LTD-DX and LFU/LTD-EI used by CDC are total infants not passing the final hearing screening and total infants identified with a permanent hearing loss, respectively. More details about the HSFS and data definitions have been published (4,5). The reasons for being LFU/LTD listed in the HSFS include the following: the parents/family were contacted but unresponsive, unable to contact, and unknown. Cases in which the infant died, the parents refused services, or the parents moved were not classified as LFU/LTD.†

Data for this report are based on the HSFS conducted for the years 2006–2012, using aggregate jurisdiction-reported totals. Some jurisdictions did not respond to the HSFS in ≥1 years because completion of the survey is voluntary, the requested data were not available at the time of reporting, or another reason. Data for individual years and data at the jurisdictional level are available online.§ Eighty-three percent of jurisdictions responded to the survey in 2006, and 97% responded in 2012. Information was excluded if, after consultation with a jurisdictional EHDI program, the reported data were found to be incomplete or derived from estimated information. Because some jurisdictions did not respond to the survey in ≥1 years, there are differences in the number of jurisdictions reporting each year.

In 2012, an average of 96.6% of newborns were screened for hearing loss compared with 95.2% in 2006 (Tables 1 and 2). Overall, the number and average percentage of those infants that did not pass the hearing screening and were subsequently diagnosed with a permanent hearing loss increased from 4.8% (3,261) to 10.3% (5,475). The proportion of infants identified with hearing loss increased from 1.1 to 1.6 per 1,000 infants screened (Figure). For those infants with a confirmed, permanent hearing loss, an average of 61.7% were documented as receiving EI in 2012 compared with 55.4% in 2006 (Tables 1 and 2). The average percentage of LFU/LTD-DX decreased from 47.7% to 35.9%, and the average percentage of LFU/LTD-EI decreased from 40.3% to 24.6% (Figure).

Based on available data from the HSFS, a number of jurisdictions have made progress in documenting the diagnosis of infants with permanent hearing loss and their enrollment in EI. For example, 10 jurisdictions had an improvement of at least 10% for diagnosed hearing loss among infants who did not pass the hearing screening (Tables 1 and 2). Seventeen jurisdictions had at least a 10% improvement in infants enrolled in EI. In addition, 12 jurisdictions had a 30% decrease in LFU/LTD-DX, and 12 jurisdictions had at least a 30% decrease in their LFU/LTD-EI rates.

## Discussion

Improvements in the provision and documentation of EHDI services between 2006 and 2012 have resulted in decreases in the rate of infants referred from screening and increases in infants receiving the testing needed to confirm a hearing loss. This progress has helped drive increases in the number of children reported with permanent hearing loss from 3,261 (2006) to 5,475 (2012) and an increase in prevalence from 1.1 to per 1.6 per 1,000 screened. The increase in documented cases was accompanied by a decrease in LFU/LTD-DX of 11.8% between 2006 and 2012. Similarly, the documented receipt of EI services increased by 6.3% while LFU/LTD-EI decreased by 15.7%. Other factors that contributed at least in part to this progress include 1) improvements in the functionality of state and territorial EHDI information systems, 2) increased awareness among health care providers about the importance of documenting the receipt of follow-up services, 3) continued progress by state and territorial EHDI programs in tracking infants needing follow-up services, and 4) active support by national agencies and organizations.

To build on the progress already made in diagnosing and enrolling infants with hearing loss in EI services, continued work is needed to further reduce the number of infants classified as LFU/LTD each year. Unless infants with hearing loss receive recommended diagnostic and EI services, they are still at risk for avoidable delays in their speech and language development (2,3). In addition, without appropriate documentation, it is difficult to ensure infants are receiving recommended services. Additional coordination among audiologists, physicians, jurisdictional EHDI, and EI programs can further improve documentation and provision of services.

This report updates an earlier summary of EHDI data during 1999–2007 that provided information on infants with hearing loss (4). Since that time, there have been several important policy and practice changes that could have had a direct impact on rates of LFU/LTD. For example, some hospitals and EHDI programs now assist parents in making appointments for follow-up testing and calling families to remind them about upcoming appointments. These and other changes were developed during a collaborative improvement project funded by the Health Resources and Services Administration. All jurisdictions participated in this project and worked to develop strategies specific to their jurisdiction to increase the rates of documented follow-up testing and enrollment in EI services.¶

The findings in this report are subject to at least five limitations. First, some states and territories either did not respond to the HSFS or were only able to provide limited data in ≥1 reporting years. As a result there are differences in the number of jurisdictions reporting data each year. Second, the data reported only reflect those services that infants were documented to have received. Because reporting of newborn hearing screening and follow-up data are not required in each state and territory, it is possible for a jurisdiction to have a higher percentage of infants receiving diagnostic and EI services (and therefore lower rates of LFU/LTD) than what was reported by the HSFS. Third, there are multiple ways to calculate LFU/LTD, and the CDC definition might not fully reflect the progress jurisdictions have made in ensuring that infants receive recommended follow-up services. Fourth, there is variation between jurisdictions in the percentage diagnosed with permanent hearing loss and the reasons for this, including the impact of different screening protocols, cannot be assessed with currently available HSFS data. Fifth, all HSFS data are reported voluntarily and might include inaccuracies because some jurisdictions did not correctly report LFU/LTD and other data in accordance with the HSFS data definitions.

To build on the recent improvements summarized here and ensure continued progress toward identifying and providing EI for all infants with permanent hearing loss, current practices should evolve and take advantage of new collaborations and opportunities, such as emerging technologies. Improvements in existing clinical and public health infrastructures and adoption of technologies, such as electronic health records and clinical decision support tools, can assist providers and EHDI programs in improving coordination, delivery, and documentation of recommended EHDI services (6–9).

What is already known on this topic?

Progress has been made in screening and diagnosing infants with hearing loss, reducing the number of infants lost to follow-up/lost to documentation, and increasing enrollment in early intervention. Ensuring infants receive recommended services is crucial to help prevent delays in speech, language, social, and emotional development that can occur when permanent hearing loss is not identified early.

What is added by this report?

Analysis of Early Hearing Detection and Intervention program survey data showed that, during 2006–2012, the number of jurisdictions reporting data increased from 49 to 57, rates of screening increased from 95.2% to 96.6%, rates of diagnosis among infants not passing the final screening increased from 4.8% to 10.3%, and enrollment in early intervention of infants diagnosed with permanent hearing loss increased from 55.4% to 61.7%, while the rates of lost to follow-up/lost to documentation declined.

What are the implications for public health practice?

EHDI programs should continue to work with health care providers who provide diagnostic and early intervention services to accurately document the receipt of necessary follow-up services, thereby increasing the opportunities for infants to receive proper care to minimize the negative impact that hearing loss can have on their speech, language, and emotional development.

## Figures and Tables

**FIGURE:**
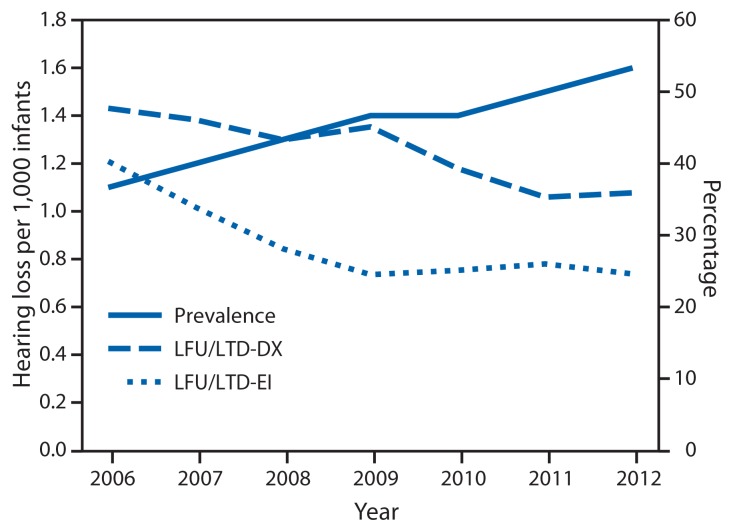
Prevalence of infants identified with hearing loss and percentage of those infants who were lost to follow-up/lost to documentation (LFU/LTD) for diagnostic testing (DX) or for early intervention (EI) — United States, 2006–2012

**TABLE 1:** Number and percentages of infants screened, diagnosed, and enrolled in early intervention programs for hearing loss, by jurisdiction and birth year — United States, 2006*

Jurisdiction†	2006										

	Screening		Diagnosis					Early intervention			
		
	Screened	Not pass screening	Permanent hearing loss			LFU/LTD-DX		Enrolled		LFU/LTD-EI	
					
	(%)	No.	No.	%	Prevalence per 1,000 screened	No.	(%)	No.	(%)	No.	(%)
Alabama	(97.6)	2,699	43	(1.6)	0.7	572	(21.2)	32	(74.4)	11	(25.6)
Alaska	(90.4)	301	21	(7.0)	2.1	224	(74.4)	0	—	21	(100.0)
American Samoa	—	—	—	—	—	—	—	—	—	—	—
Arizona	(96.3)	1,982	107	(5.4)	1.1	1,722	(86.9)	98	(91.6)	2	(1.9)
Arkansas	(97.1)	948	45	(4.7)	1.2	590	(62.2)	12	(26.7)	32	(71.1)
California	—	—	—	—	—	—	—	—	—	—	—
Colorado	(98.0)	192	115	(59.9)	1.7	17	(8.9)	115	(100.0)	0	—
CNMI	98.9)	38	2	(5.3)	1.4	27	(71.1)	2	(100.0)	0	—
Connecticut	(99.0	383	62	(16.2)	1.5	41	(10.7)	40	(64.5)	14	(22.6)
Delaware	—	—	—	—	—	—	—	—	—	—	—
DC	(99.3)	241	8	(3.3)	0.5	0	—	8	(100.0)	0	—
Florida	(87.9)	2,655	185	(7.0)	0.8	2,470	(93.0)	121	(65.4)	55	(29.7)
Georgia	(97.5)	5,326	52	(1.0)	0.4	5,271	(99.0)	31	(59.6)	19	(36.5)
Guam	(83.8)	119	8	(6.7)	2.8	104	(87.4)	3	(37.5)	2	(25.0)
Hawaii	(98.6)	255	62	(24.3)	3.3	75	(29.4)	49	(79.0)	2	(3.2)
Idaho	(99.1)	1,039	30	(2.9)	1.3	63	(6.1)	28	(93.3)	0	—
Illinois	—	—	—	—	—	—	—	—	—	—	—
Indiana	(97.8)	1,665	112	(6.7)	1.3	248	(14.9)	—	—	—	—
Iowa	(97.7)	1,944	73	(3.8)	1.9	0	—	—	—	—	—
Kansas	(96.6)	1,196	68	(5.7)	1.7	0	—	12	(17.6)	54	(79.4)
Kentucky	(99.3)	2,193	33	(1.5)	0.6	1,348	(61.5)	23	(69.7)	10	(30.3)
Louisiana	(95.9)	1,617	34	(2.1)	0.6	1,484	(91.8)	18	(52.9)	15	(44.1)
Maine	(96.6)	305	13	(4.3)	1.0	194	(63.6)	0	—	13	(100.0)
Marshall Islands	—	—	—	—	—	—	—	—	—	—	—
Maryland	(94.8)	3,620	108	(3.0)	1.5	3,369	(93.1)	0	—	108	(100.0)
Massachusetts	(98.9)	1,299	226	(17.4)	2.9	93	(7.2)	152	(67.3)	54	(23.9)
Michigan	(98.0)	1,882	101	(5.4)	0.8	1,324	(70.4)	33	(32.7)	68	(67.3)
Micronesia	—	—	—	—	—	—	—	—	—	—	—
Minnesota	(82.6)	2,695	65	(2.4)	1.1	2,601	(96.5)	29	(44.6)	36	(55.4)
Mississippi	(98.6)	541	70	(12.9)	1.6	41	(7.6)	40	(57.1)	16	(22.9)
Missouri	(98.4)	1,387	35	(2.5)	0.4	509	(36.7)	25	(71.4)	9	(25.7)
Montana	(93.0)	392	17	(4.3)	1.5	374	(95.4)	0	—	17	(100.0)
Nebraska	(98.9)	181	28	(15.5)	1.1	104	(57.5)	16	(57.1)	12	(42.9)
Nevada	—	—	—	—	—	—	—	—	—	—	—
New Hampshire	(98.7)	318	58	(18.2)	4.2	188	(59.1)	33	(56.9)	25	(43.1)
New Jersey	(98.9)	1,876	102	(5.4)	0.9	1,454	(77.5)	69	(67.6)	30	(29.4)
New Mexico	(71.5)	1,342	38	(2.8)	1.9	0	—	37	(97.4)	0	—
New York	(98.9)	—	—	—	—	—	—	—	—	—	—
North Carolina	(98.2)	1,505	234	(15.5)	1.8	808	(53.7)	146	(62.4)	88	(37.6)
North Dakota	(96.6)	424	6	(1.4)	0.8	397	(93.6)	0	—	6	(100.0)
Ohio	—	—	—	—	—	—	—	—	—	—	—
Oklahoma	(95.0)	1,875	81	(4.3)	1.6	468	(25.0)	70	(86.4)	7	(8.6)
Oregon	(38.6)	930	78	(8.4)	4.2	359	(38.6)	53	(67.9)	17	(21.8)
Palau	(74.1)	—	—	—	—	—	—	—	—	—	—
Pennsylvania	(95.5)	1,400	143	(10.2)	1.0	290	(20.7)	143	(100.0)	0	—
Rhode Island	(98.9)	141	15	(10.6)	1.2	17	(12.1)	12	(80.0)	2	(13.3)
South Carolina	(98.3)	1,911	77	(4.0)	1.3	509	(26.6)	56	(72.7)	21	(27.30
South Dakota	(97.8)	427	4	(0.9)	0.3	381	(89.2)	0	—	4	(100.0)
Tennessee	(89.9)	3,499	50	(1.4)	0.6	1,297	(37.1)	28	(56.0)	15	(30.0)
Texas	(98.7)	7,656	259	(3.4)	0.7	487	(6.4)	0	—	259	(100.0)
Utah	(98.4)	731	56	(7.7)	1.1	414	(56.6)	33	(58.9)	20	(35.7)
Vermont	(96.3)	59	9	(15.3)	1.5	29	(49.2)	5	(55.6)	4	(44.4)
Virginia	(97.6)	2,318	132	(5.7)	1.3	486	(21.0)	93	(70.5)	21	(15.9)
Washington	(93.9)	2,302	119	(5.2)	1.5	1,731	(75.2)	0	—	119	(100.0)
West Virginia	(96.0)	67	11	(16.4)	0.5	3	(4.5)	6	(54.5)	3	(27.3)
Wisconsin	(93.9)	1,586	52	(3.3)	0.8	0	—	24	(46.2)	28	(53.8)
Wyoming	(98.6)	28	14	(50.0)	2.0	6	(21.4)	8	(57.1)	0	—
**Totals**	**(95.2)**	**67,490**	**3,261**	**(4.8)**	**1.1**	**32,189**	**(47.7)**	**1,703**	**(55.4)**	**1,239**	**(40.3)**

**Abbreviations:** CNMI = Commonwealth of Northern Mariana Islands; DC = District of Columbia; LFU/LTD-DX = lost to follow-up/lost to documentation for diagnostic testing; LFU/LTD-EI = lost to follow-up/lost to documentation for early intervention.

**Source:** The Early Hearing Detection and Intervention program’s Hearing Screening and Follow-up Survey.

* Some jurisdictions did not provide complete data.

† More comparisons can be made using interactive maps at http://ehdidash.cdc.gov/IAS_WebApp/.

**TABLE 2:** Number and percentages of infants screened, diagnosed, and enrolled in early intervention programs for hearing loss, by jurisdiction and birth year — United States, 2012*

Jurisdiction†	2012										

	Screening		Diagnosis					Early intervention			
		
	Screened	Not pass screening	Permanent hearing loss			LFU/LTD-DX		Enrolled		LFU/LTD-EI	
					
	(%)	No.	No.	(%)	Prevalence per 1,000 screened	No.	(%)	No.	(%)	No.	(%)
Alabama	(98.5)	222	60	(27.0)	1.1	86	(38.7)	35	(58.3)	10	(16.7)
Alaska	(95.8)	159	22	(13.8)	2.1	72	(45.3)	11	(50.0)	6	(27.3)
American Samoa	(99.2)	10	1	(10.0)	0.9	4	(40.0)	0	—	0	—
Arizona	(98.8)	833	157	(18.8)	1.8	410	(49.2)	61	(38.9)	8	(5.1)
Arkansas	(95.4)	743	40	(5.4)	1.1	248	(33.4)	13	(32.5)	10	(25.0)
California	(95.9)	2,770	945	(34.1)	2.0	436	(15.7)	718	(76.0)	54	(5.7)
Colorado	(97.9)	716	116	(16.2)	1.8	538	(75.1)	55	(47.4)	35	(30.2)
CNMI	(97.8)	27	3	(11.1)	2.7	15	(55.6)	3	(100.0)	0	—
Connecticut	(98.9)	579	50	(8.6)	1.4	202	(34.9)	35	(70.0)	13	(26.0)
Delaware	(99.0)	209	20	(9.6)	1.8	101	(48.3)	0	—	20	(100.0)
DC	(86.3)	374	25	(6.7)	2.1	52	(13.9)	21	(84.0)	4	(16.0)
Florida	(97.3)	1,625	225	(13.8)	1.1	736	(45.3)	167	(74.2)	34	(15.1)
Georgia	(97.3)	1,115	229	(20.5)	1.8	491	(44.0)	143	(62.4)	28	(12.2)
Guam	(99.1)	25	9	(36.0)	2.9	3	(12.0)	8	(88.9)	0	—
Hawaii	(98.3)	221	54	(24.4)	2.9	33	(14.9)	36	(66.7)	9	(16.7)
Idaho	(99.3)	720	64	(8.9)	3.0	226	(31.4)	62	(96.9)	1	(1.6)
Illinois	(99.4)	—	—	—	—	—	—	198	(81.5)	44	(18.1)
Indiana	(96.6)	2,364	145	(6.1)	1.8	257	(10.9)	83	(57.2)	48	(33.1)
Iowa	(98.7)	461	48	(10.4)	1.3	127	(27.5)	36	(75.0)	9	(18.8)
Kansas	(98.7)	354	93	(26.3)	2.3	42	(11.9)	67	(72.0)	17	(18.3)
Kentucky	(99.6)	2,344	58	(2.5)	1.1	240	(10.2)	38	(65.5)	20	(34.5)
Louisiana	(98.9)	3,404	66	(1.9)	1.1	1,073	(31.5)	43	(65.2)	15	(22.7)
Maine	(97.9)	208	23	(11.1)	1.9	33	(15.9)	13	(56.5)	8	(34.8)
Marshall Islands	(52.1)	47	2	(4.3)	4.3	39	(83.0)	0	—	2	(100.0)
Maryland	(99.4)	820	78	(9.5)	1.1	257	(31.3)	49	(62.8)	24	(30.8)
Massachusetts	(99.1)	1,153	200	(17.3)	2.8	29	(2.5)	143	(71.5)	18	(9.0)
Michigan	(99.0)	1,173	162	(13.8)	1.5	569	(48.5)	32	(19.8)	125	(77.2)
Micronesia	(91.6)	—	—	—	—	—	—	—	—	—	—
Minnesota	(98.1)	601	162	(27.0)	2.4	150	(25.0)	83	(51.2)	48	(29.6)
Mississippi	(98.9)	492	76	(15.4)	2.0	26	(5.3)	58	(76.3)	9	(11.8)
Missouri	(97.9)	1,431	100	(7.0)	1.3	461	(32.2)	66	(66.0)	7	(7.0)
Montana	(96.3)	193	14	(7.3)	1.2	94	(48.7)	7	(50.0)	5	(35.7)
Nebraska	(99.4)	120	36	(30.0)	1.4	34	(28.3)	30	(83.3)	2	(5.6)
Nevada	(95.8)	340	41	(12.1)	1.2	174	(51.2)	34	(82.9)	3	(7.3)
New Hampshire	(97.8)	356	13	(3.7)	1.1	129	(36.2)	12	(92.3)	0	—
New Jersey	(99.4)	883	129	(14.6)	1.3	378	(42.8)	92	(71.3)	24	(18.6)
New Mexico	(66.6)	911	46	(5.0)	2.6	693	(76.1)	37	(80.4)	9	(19.6)
New York	(83.2)	—	—	—	—	—	—	—	—	—	—
North Carolina	(99.1)	854	190	(22.2)	1.6	323	(37.8)	161	(84.7)	13	(6.8)
North Dakota	(98.8)	369	24	(6.5)	2.1	182	(49.3)	24	(100.0)	0	—
Ohio	(98.6)	3,945	213	(5.4)	1.5	1,254	(31.8)	129	(60.6)	68	(31.9)
Oklahoma	(99.0)	2,386	74	(3.1)	1.5	592	(24.8)	57	(77.0)	17	(23.0)
Oregon	(96.3)	1,287	82	(6.4)	1.9	624	(48.5)	56	(68.3)	18	(22.0)
Palau	(99.3)	4	0	—	0.0	2	(50.0)	—	—	—	—
Pennsylvania	(95.6)	2,270	206	(9.1)	1.5	176	(7.8)	162	(78.6)	16	(7.8)
Rhode Island	(99.4)	116	12	(10.3)	1.0	24	(20.7)	11	(91.7)	0	—
South Carolina	(96.9)	775	85	(11.0)	1.6	388	(50.1)	40	(47.1)	45	(52.9)
South Dakota	(98.1)	280	27	(9.6)	2.2	234	(83.6)	0	—	27	(100.0)
Tennessee	(97.9)	3,585	84	(2.3)	1.0	1,239	(34.6)	73	(86.9)	9	(10.7)
Texas	(98.8)	4,927	412	(8.4)	1.1	3,776	(76.6)	70	(17.0)	241	(58.5)
Utah	(98.9)	696	100	(14.4)	1.9	381	(54.7)	70	(70.0)	19	(19.0)
Vermont	(99.9)	155	3	(1.9)	0.5	46	(29.7)	2	(66.7)	0	—
Virginia	(98.4)	1,100	161	(14.6)	1.6	407	(37.0)	110	(68.3)	48	(29.8)
Washington	(95.0)	988	154	(15.6)	1.9	495	(50.1)	0	—	154	(100.0)
West Virginia	(85.7)	597	8	(1.3)	0.4	310	(51.9)	4	(50.0)	4	(50.0)
Wisconsin	(99.1)	577	110	(19.1)	1.7	85	(14.7)	54	(49.1)	56	(50.9)
Wyoming	(96.3)	47	18	(38.3)	2.7	10	(21.3)	15	(83.3)	0	—
**Totals**	**(96.6)**	**52,961**	**5,475**	**(10.3)**	**1.6**	**19,006**	**(35.9)**	**3,527**	**(61.7)**	**1,404**	**(24.6)**

**Abbreviations:** CNMI = Commonwealth of Northern Mariana Islands; DC = District of Columbia; LFU/LTD-DX = lost to follow-up/lost to documentation for diagnostic testing; LFU/LTD-EI = lost to follow-up/lost to documentation for early intervention.

**Source:** The Early Hearing Detection and Intervention program’s Hearing Screening and Follow-up Survey.

* Some jurisdictions did not provide complete data.

† More comparisons can be made using interactive maps at http://ehdidash.cdc.gov/IAS_WebApp/.

## Footnotes

* **Lost to follow-up (LFU)** describes an event in which an infant needs a specific follow-up action but does not receive it. LFU for diagnosis (LFU-DX) occurs when an infant does not pass the hearing screening, is referred for diagnostic testing by a qualified provider (e.g., an audiologist) but never receives the testing needed to confirm whether a hearing loss is present. LFU for early intervention (LFU-EI) occurs when an infant diagnosed with a permanent hearing loss is not enrolled in any early intervention services. **Lost to documentation (LTD)** describes an event in which an infant receives a specific follow-up action, but neither confirmation that the follow-up was provided nor the results are reported to the jurisdictional EHDI program. LTD for diagnosis (LTD-DX) occurs when an infant not passing the hearing screening does receive follow-up testing that either confirms a hearing loss or does not identify any loss but this information is not reported to the jurisdictional EHDI program. LTD for early intervention (LTD-EI) occurs when an infant diagnosed with a permanent hearing loss is enrolled in early intervention services but this information is not reported to the jurisdictional EHDI program.

† In 2006, of those infants needing diagnostic follow-up testing, 998 (2.2%) did not receive it because of parent refusals or infant deaths and 510 (1.1%) because of being nonresidents or moving out of state. In 2012, of those infants needing diagnostic follow-up testing, 2,141 (4.0%) did not receive it because of parent refusals or infant deaths and 1,505 (2.8%) because of being nonresidents or moving out of state.

§ Available at http://www.cdc.gov/ncbddd/hearingloss/ehdi-data.html.

¶ Additional information available at http://newbornhearing.nichq.org/solutions/ihsis.
